# Isolation and Characterization of Nodule-Associated *Exiguobacterium sp*. from the Root Nodules of *Fenugreek* (*Trigonella foenum-graecum*) and Their Possible Role in Plant Growth Promotion

**DOI:** 10.1155/2012/693982

**Published:** 2012-02-23

**Authors:** Geetha Rajendran, Maheshwari H. Patel, Sanket J. Joshi

**Affiliations:** ^1^Ashok and Rita Patel Institute of Integrated Study and Research in Biotechnology and Allied Sciences (ARIBAS), Sardar Patel University, V. V. Nagar, Gujarat, Anand 388120, India; ^2^Department of Biology, College of Science, Sultan Qaboos University, P. B. No. 36, Muscat, 123, Oman

## Abstract

One of the ways to increase the competitive survivability of rhizobial biofertilizers and thus achieve better plant growth under such conditions is by modifying the rhizospheric environment or community by addition of nonrhizobial nodule-associated bacteria (NAB) that cause better nodulation and plant growth when coinoculated with rhizobia. A study was performed to investigate the most commonly associated nodule-associated bacteria and the rhizospheric microorganisms associated with the *Fenugreek* (*Trigonella foenum-graecum*) plant. Isolation of nonrhizobial isolates from root nodules of *Fenugreek* was carried out along with the rhizospheric isolates. About 64.7% isolates obtained from *Fenugreek* nodules were gram-negative coccobacilli, 29.41% were gram-positive bacilli, and all rhizospheric isolates except one were gram-positive bacilli. All the isolates were characterized for their plant growth promoting (PGP) activities. Two of the NAB isolates M2N2c and B1N2b (*Exiguobacterium sp.*) showed maximum positive PGP features. Those NAB isolates when coinoculated with rhizobial strain—*S. meliloti*, showed plant growth promotion with respect to increase in plant's root and shoot length, chlorophyll content, nodulation efficiency, and nodule dry weight.

## 1. Introduction

Plant growth promoting rhizobacteria (PGPR) are a very small portion (about 2–5%) of the total rhizobacterial community [1]. Such PGPR use one or more direct or indirect mechanisms to improve the growth and health of plants. These mechanisms can be active simultaneously or independently at different stages of plant growth. Among these are phosphate solubilization, biological nitrogen fixation, improvement of other plant nutrients uptake, and phytohormone production (like indole-3-acetic acid) are some of the regulators that profoundly influence plant growth [2]. Moreover, biological control of plant pathogens and deleterious microbes, through the production of antibiotics, lytic enzymes, hydrogen cyanide, and siderophores or through competition for nutrients and space can improve significantly the plant health and promote growth [1]. 

The leguminous plants are symbiotically associated with the rhizobia and this requires active nitrogen fixation and the interaction plays a key role in the agricultural crop production. Enhancement of legume nitrogen fixation by coinoculation of rhizobia with plant growth promoting rhizobacteria (PGPR) is a way to improve nitrogen availability in sustainable agriculture production systems. Many PGPRs are known to promote plant growth directly by the production of plant growth regulators and improvements in plant nutrient uptake [3, 4] or indirectly by the production of metabolites like antibiotics, siderophores, and so forth that decrease the growth of phytopathogens [3]. PGPR also have beneficial effects on legume growth and some strains enhance legume nodulation and nitrogen fixation by affecting interaction between plant and rhizobia [5]. Many studies have shown that simultaneous infection with rhizobia and rhizospheric bacteria increases nodulation and growth in a wide variety of legumes [6–9]. Such nodule-assisting bacteria may be free-living rhizobacteria or endophytic. Endophytic bacteria reside intercellularly or intracellularly within host tissues [10] and therefore are at advantage as compared to free-living counterparts by being protected from environmental stresses and microbial competition [11]. Depending on their effect on the host plant, endophytic bacteria can be categorized into three groups: plant growth promoting, plant growth inhibiting, and plant growth neutral [10]; however a major proportion of bacterial endophytes have plant growth promoting effect [12].

The presence of bacteria (*Agrobacterium radiobacter*) other than *Rhizobium* in root nodules was first reported by Sturz et al. [13]. Manninger and Antal [14] also reported rhizobia and other bacteria in the root nodules of the *Leguminosae*. Endophytic bacteria have also been isolated from legume plants such as alfalfa [15], clover [13], and soyabean [16]. Bacteria of several genera have been isolated from legume tissues, including *Aerobacter, Pseudomonas, Agrobacterium, Bacillus, Chryseomonas, Curtobacterium, Enterobacter, Erwinia, Flavimonas, *and* Sphingomonas* [13, 15]. Majority of the recent studies on legume-rhizobacterial interaction have been confined to soyabean (*Glycine max L*), cowpea (*Vigna unguiculata L*), chickpea (*Cicer arietinum L*), the common bean (*Phaseolus vulgaris L*), and red clover (*Trifolium pretense L*). Coinoculation of other PGPR with rhizobia is envisaged as an important practice in the development of sustainable agriculture. Available reports indicate improved plant yield and plant health under greenhouse conditions with respect to increase in root wet weight and nodulation when coinoculated with nodule endophytes, compared to inoculation with rhizobia alone [13, 17, 18]. PGPB that have been coinoculated with rhizobia include strains of the following well-known rhizobacteria: *Azospirillum *[9], *Azotobacter* [19], *Bacillus* [20], and *Pseudomonas *[21]. Coinoculation of some *Pseudomonas* and *Bacillus *strains along with effective *Rhizobium sp*. is shown to stimulate chickpea growth, nodulation, and nitrogen fixation [5]. Some *Serratia* strains, such as *S. proteamaculans* 1-102 and *S. liquefaciens* 2-68, have beneficial effects on legume plant growth [21, 22].

*Bacilli* are spore-forming, gram-positive, rod-shaped bacteria that comprise one of the most common soil bacterial groups and they are frequently isolated from the rhizosphere of plants. *Bacillus *species are also common endophytes [11, 23, 24]. Due to their spore-forming capability they are readily adaptable to field applications [23]. *Exiguobacterium sp. *fall in the class *Bacilli*, order *Bacillales* and family *Bacillaceae*. They are non-spore-forming motile gram-positive bacilli. Our lab work has shown the plant growth promotion of *Cajanus cajan* (Pigeon pea) in the presence of nodule-associated *Bacillus sp. *when applied in the pot conditions in conjunction with the rhizobial biofertilizers strain. The present work deals with the study of such interactions and their effect on the plant health and nodulation in *Feenugreek* (*Trigonella foenum-graecum*) plant.

## 2. Materials and Methods

### 2.1. Isolation of Bacterial Strains

Endophytes and epiphytes were isolated from root nodules of *Fenugreek* (*Trigonella foenum-graecum*) plant. Nodules were taken from freshly uprooted plants. Roots of the plant were thoroughly washed under tap water to remove the mud and soil particles. Healthy and pink nodules were selected for the isolation of nodule-associated bacteria (NAB). Nodules were safely cut from the root and were washed under running tap water and then for 30 sec in 70% ethanol solution. They were then treated with 0.1% HgCl_2 _for 2 min and successively washed three times with sterile distilled water under aseptic condition for 1 min each. The nodules were put in 1.5 mL microfuge tubes containing 0.5 mL N-saline. Then the nodules were crushed with the help of sterile forceps and the 100 *μ*L contents were spread on Nutrient Agar (NA) plate. The 100 *μ*L of the third wash of sterile distilled water was taken and spread on NA plates for sterility checking. All the plates were incubated at 28 ± 2°C for 24 h. Colonies were picked after 24 h. The cultures were maintained on NA slants with regular subculturing [25].

### 2.2. Culture Conditions

All the NAB strains were maintained on Yeast Extract Mannitol Agar (YEMA) as well as NA and the rhizospheric strains were maintained on NA plates and grown on Luria Bertani (LB) Broth. All the isolates were checked for their colony morphologies, Gram's nature, and motility.

### 2.3. Characterization of the Plant Growth Promoting Properties of Bacterial Isolates

Potential plant growth promoting abilities of all the isolated strains were checked by studying their ability to produce siderophores, phosphate solubilisation, and Indole acetic acid (IAA) production. All the isolates were also tested for their antifungal activity against *Fusarium oxysporum*, *Alternaria burnsii*, *Trichoderma reesei*, *Rhizoctonia solani, Chrysosporium indicum, *and *Rhizoctonia bataticola* as reported [26]. All the isolates were tested for their IAA production as follows: loopful of each culture was inoculated in LB (2 mL) containing 50 *μ*g/mL tryptophan and incubated at 28°C for 24 h on shaking condition, centrifuged at 9000 rpm for 15 min. Two mL of supernatant was taken in fresh tube and 2-3 drops of orthophosphoric acid was added, to this 4 mL of FeCl_3_-HClO_4_ reagent (1 mL of 0.5 M FeCl_3_ in 50 mL of 35% HClO_4_) was added. After 25 min incubation at room temperature, absorbance was measured at 530 nm. Auxin quantification values were recorded by preparing standard calibration curve made by using IAA standard in the range of 10–100 *μ*g/mL. IAA stock solution was prepared as 100 *μ*g/mL in 50% ethanol. Standard graph of IAA concentration was plotted against OD_530_ and the concentration of IAA in samples was calculated [27].

### 2.4. Siderophore Cross-Utilization Test

The standard rhizobial strain* S. meliloti *was tested for its ability to utilize siderophores produced by all the isolates according to Khan et al. [28].

### 2.5. Coinoculation Studies

The growth profiles of* S. meliloti* were compared in presence of the M2N2c and B1N2b under deferrated (iron from the media was removed by extracting it twice with a solution of 2% (w/v) 8-hydroxy quinolone in chloroform) conditions in LB, by adding 0.5 mM EDTA. For this study the freshly grown isolates (10^2^ cfu/mL) in LB were used. Both M2N2c and B1N2b (10^2^ cfu/mL) were coinoculated separately with freshly grown *S. meliloti* (10^2^ cfu/mL) in LB containing 0.5 mM EDTA and were incubated at 28 ± 2°C at 180 rpm. Aliquots were collected at a regular interval, serially diluted, and plated on LB agar plates, to get well-isolated colonies. The plates were incubated at 28 ± 2°C for 48–72 h and the cfu/mL of *S. meliloti*, M2N2c and B1N2b was estimated. Since M2N2c, B1N2b, and *S. meliloti *had different colony characteristics it was easy to enumerate them without marking the strains. Appropriate controls of individual culture without coinoculation were also kept along with the experimental sets in order to compare their growth pattern. All the coinoculation experiments were performed in triplicates.

### 2.6. Plant Inoculation Studies with NAB

The plant inoculation studies with *Fenugreek* were carried out as mentioned in Rajendran et al. [26]. The germinated *Fenugreek* seeds were inoculated with rhizobial strain *S. meliloti* and the culture-coated seedlings were sown in autoclaved soil systems consisting of the following: autoclaved soil (Typic Ustochrepts with a Sandy-loam type of structure having good fertility status; pH 7.3) sown with seedlings without any bacterization as the negative control (NC); seedlings with* S. meliloti* alone as a positive control (PC); M2N2c and B1N2b alone individually as the NAB control (NABC) and seedlings coinoculated with *S. meliloti *and M2N2c or B1N2b as the experimental systems. All the pot experiments were performed in triplicates and plants were harvested after 55 days of inoculation when well-developed nodules could be detected and various parameters such as shoot length, root length, root to shoot ratio, chlorophyll content, and nodule number per plant and average nodule weight per plant were monitored.

### 2.7. Chlorophyll Estimation

Midrib of the leaves was removed and one gram leaf tissue was crushed in 80% acetone; chlorophyll was measured spectrophotometrically using the specific absorption coefficients for “chlorophyll a” at 664 nm and “chlorophyll b” at 647 nm. Chlorophyll content was estimated according to Graan and Ort [29].

### 2.8. Identification of the NAB Isolates (M2N2c and B1N2b)

Both the NAB isolates were identified by amplifying their 16S rRNA gene using the universal eubacterial forward primer 27F (5′ GAG AGT TTG ATC CTG GCT CAG 3′) and reverse primer 1107R (5′ GCT CGT TGC GGG ACT TAA CC 3′). The partial length 16S rRNA amplicon was sequenced. The sequencing was carried out at Bangalore Genei Pvt. ltd., India. The sequence obtained from B1N2b and M2N2c was matched with the nucleotide database available at the Gene Bank, using BLAST tool at NCBI.

### 2.9. Statistical Analysis

The statistical analysis of the results obtained was done by one way ANOVA (analysis of variance) using the web trial version of SigmaStat 3.5. The difference between all the comparisons made is significant at 95% confidence interval.

## 3. Results

Root nodule associated bacteria were isolated from the nodules of *Fenugreek *plants from different field areas on the YEMA/NA and rhizospheric isolates from the soil adhering to the roots of the plant were isolated on the Nutrient Agar (NA) medium. Colonies showing different morphological characteristics on the plates were selected for further characterization. A total 24 (17 NAB + 07 rhizospheric) strains were isolated with different morphological characteristics and their PGPR characteristics were studied. Majority (64.7%) of the nodule associated bacteria (NAB) in our studies belonged to gram negative coccobacilli strains followed by 29.4% being gram positive bacilli and only 5.8% being positive cocci. Whereas, all the rhizospheric bacteria except R2 (gram negative coccobacilli) were gram positive bacilli (Table 1). Only 35% of the NAB was motile and again all rhizospheric except R2 were non-motile (data not shown). When tested for the siderophore production it was observed that all the isolates except C2 showed catecholate siderophore production. M2N2c showed maximum catecholate production 907.7 *μ*g/mL and minimum hydroxamate of 39.2 *μ*g/mL. Seventy percent of NAB produced hydroxamate but the amount produced was very low except B1N3 which showed 103 *μ*g/mL. Among the NAB maximum hydroxamate production was shown by M2N1b (136.2 *μ*g/mL). Seventy percent of the rhizospheric isolates showed hydroxamate siderophore production above 100 *μ*g/mL with an exception of C1 and C3 whereas none of the NAB′s showed such characteristic except M2N1b and B1N3 (Table 1). The highest hydroxamate producer was C2 (156.8 *μ*g/mL) and it did not produce catecholate at all. The entire NAB as well as rhizospheric isolates showed a positive IAA production. Maximum IAA production was shown by M2EP2 (62.64 *μ*g/mL) (Table 1). Phosphate solubilization was shown by only three NAB isolates M2N2c, M2EP3 and B1N2b whereas all the rhizospheric isolates except C1 and R2 showed phosphate solubilization. Only 29% of the total isolate showed organic acid production which included M2EP1, M2EP2, M2EP3, M2N2c and B1N2b among NAB and R3 and R4 among rhizospheric isolates. The entire NAB showed antifungal activity against *Alternaria *except M1EP1 which showed for* F. oxysporum *as well. All the isolates showed protease production with maximum production shown by M2N2c, B1N2b, C2 and C3. Here we found that the *S. meliloti* could cross-utilize the siderophores produced by seven NAB isolates (M2EP1, M2EP2, M2EP3, M2N2a, M2N2b, M2N2c, B1N2b) and two of the rhizospheric isolate C2 and C3 (data not shown).

**Figure 1:**
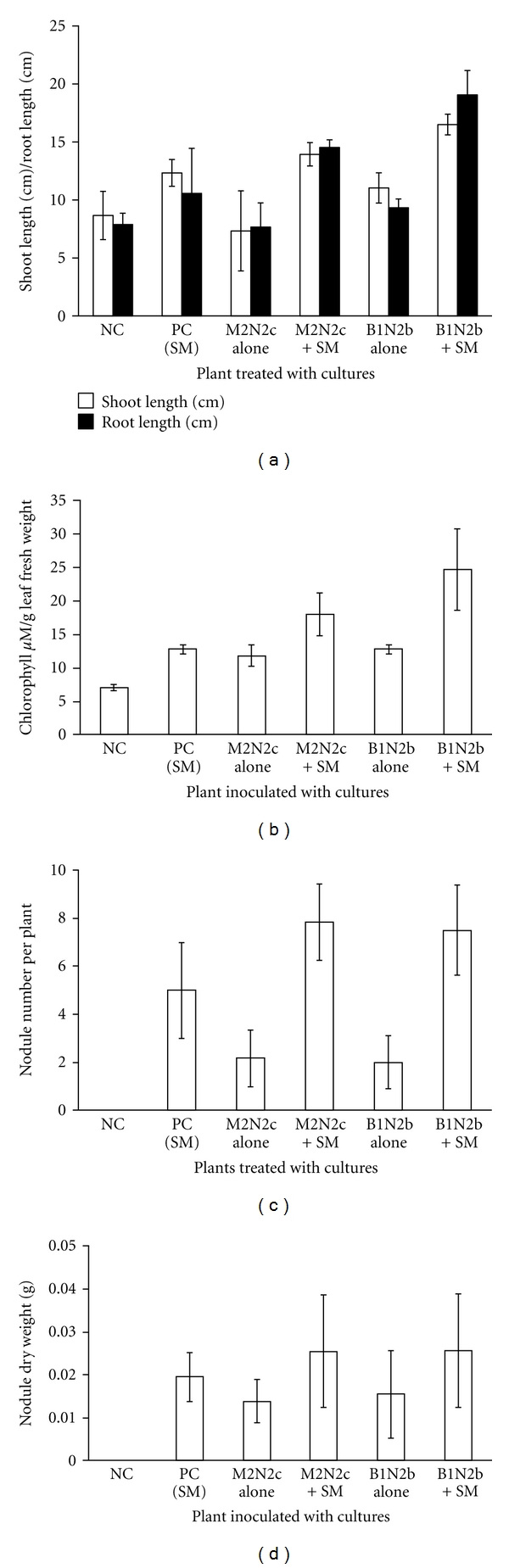
Effect of NAB isolates on the shoot length, root length (a), chlorophyll content (b), nodule number (c), and nodule dry weight (d) of the Fenugreek plants when coinoculated with *S. meliloti* under autoclaved soil conditions. The untreated plants were used as negative control (NC), plants treated with *S. meliloti,* M2N2c, and B1N2b alone were used as the positive control (PC) and NB control (NABC), respectively. All the treatments were performed in triplicates.

Since M2N2c and B1N2b among the NAB showed maximum positive PGP characteristics and also *S. meliloti* was able to cross-utilize their siderophore it was our interest to see whether the presence of these isolates affected the growth of *S. meliloti *in lab as well as the in planta studies. These isolates B1N2b and M2N2c showed 97% and 93% identity with 16S rRNA gene of *Exiguobacterium sp. *M527 (AB461812), respectively. GenBank accession numbers of 16S rRNA gene sequences of isolates *Exiguobacterium sp.* M2N2c and B1N2b are JF795956 and JF795957, respectively. Both the isolates when coinoculated with standard *S. meliloti *in the flask and it was observed that the coinoculation with the rhizobial strain stimulated the growth of *S. meliloti* as compared to the one grown alone. The isolate M2N2c caused a better stimulation in the growth of *S. meliloti* as compared to B1N2b (data not shown). The in-planta effect of the NAB isolates were checked by coinoculating them with *S. meliloti*. The M2N2c and B1N2b were tested for their nodule promoting and plant growth promoting activity. Both M2N2c and B1N2b showed a significant increase in plant's shoot length, root length (Figure 1(a)), and chlorophyll content of the leaf (Figure 1(b)). Roots allow a plant to absorb water and nutrients from the surrounding soil, and a healthy root system is the key to a healthy plant. The root to shoot ratio is one measure which helps us to assess the overall health of the plants. All the systems were compared with the control group of plants which provided us with a “normal” root to shoot ratio for each of the plant types, any changes from this normal level (either up or down) thus was used as an indication of a change in the overall health of the plant. The data were combined to get an accurate understanding of what was happening with our plant systems. An increase in root-to-shoot ratio is considered as an indication of a healthier plant, provided the increase came from greater root size and not from a decrease in shoot size. Isolates M2N2c and B1N2b when coinoculated showed an increase in root-to-shoot ratio; the increase in root-to-shoot ratio did not arise due to the decreased growth of shoot rather the growth studies showed a proportional increase in root length and shoot length as compared to the plants inoculated only with *S. meliloti* and the ones which were uninoculated, thus indicating better plant growth. There are reports stating that the plants of white clover var. Blanca under nodulated conditions always displayed a higher root/shoot ratio [30]. There was a significant increase in the average nodule number per plant when *S. meliloti* was coinoculated with M2N2c or B1N2b (Figure 1(c)) as compared to the plants which were inoculated with *S. meliloti* or NAB alone and also a considerable increase in the nodule dry weight could be seen under the coinoculated condition (Figure 1(d)).

## 4. Discussion 

The cooperative interaction between rhizobia and other plant root colonizing bacteria is of relevance in improvement of nodulation and N_2_ fixation in legume plants [31]. We describe here isolation of nonrhizobial bacteria that are closely associated with nodules of leguminous. The isolation procedure adopted involved thorough surface sterilization of nodules and was thus specifically aimed to eliminate nodule epiphytes. Sturz et al. [13] have reported isolation of 114 bacterial isolates from 15 root nodules, of which nearly 60% were rhizobia while the remainder was identified as belonging to several other genera of which eight species were exclusively found only in root nodules. According to the results reported by Rajendran et al. [26] about 10% of the surface sterilized nodules tested showed presence of endophytic nonrhizobial flora and some nodules showed more than one morphologically distinct nonrhizobial colonys. In the present study we also had similar frequency wherein we could isolate putative endophytes from approximately 15% of the nodules processed. Majority of our putative endophytes were gram-negative coccobacilli but it was interesting to note that the isolates showing maximum positive PGPR features were gram-positive bacilli. The phylogenetic analysis using the results of biochemical tests showed a vast diversity in the microbial flora among the isolated nodule-associated organisms. The isolated NAB showed 75% similarity in the biochemical features examined. There are reports stating the rhizospheric diversity but here we observed that diversity exists even amongst the organisms associated with the nodules. Probably all the organisms whose presence has a beneficial relation might get associated with the plant nodules. There are several reports depicting bacteria isolated from roots as plant growth promoting, particularly more often when coinoculated with *Rhizobium *than when applied alone [13]. The PGPR strains, *Serratia proteamaculans* 1-102 and *Serratia liquefaciens* 2-68, enhance soyabean nodulation, improve plant growth and yields while endophytic isolates *Bacillus megaterium, Bordetella avium,* and *Curtobacterium luteum* improved clover nodulation when coinoculated with *Rhizobium leguminosarum* BV *trifolii* [32]. M2N2c and B1N2b were sequenced and they turned out to be *Exiguobacterium sp.,* a non-spore-forming motile gram-positive bacilli.* Bacillus *species are also common endophytes [11, 23, 24, 26, 33, 34]. Due to the spore-forming capability of many bacilli they are readily adaptable to field applications [23]. *Bacillus* species also have been shown to have positive growth effect on rhizobial-plant interaction. Nodulation was shown to get promoted when *Rhizobium leguminosarum* BV *trifolii* was coinoculated with either *B. insolitus* or *B. brevis* [13]. *Bacillus* sp. isolated from the root nodules of soyabean-promoted plant growth when inoculated individually as well as when coinoculated with *Bradyrhizobium japonicum* showed enhancement of plant growth and nodulation [18]. *B. megaterium* B153-2-2 showed enhanced plant growth and nodulation of soybean plant when coinoculated with *B. japonicum* [23]. There are reports showing the endophytic colonization of sweet pepper (*Capsicum annuum L*) by *Bacillus sp. *[35]. 

Many different mechanisms have been proposed as the basis of nodulation enhancement by epiphytic or endophytic root-associated bacteria [31]. Using either cell-free supernatants of PGPR cultures or pure chemicals, it has been demonstrated that plant-growth-regulating substances produced by PGPR affected the nodulation and nitrogen fixation [36, 37]. Both the strains M2N2c and B1N2b were able to produce organic acid and solubilize phosphate by producing a 6 mm diameter zone of clearance on the Pikovasky's agar. Since phosphorus is an insoluble compounds and is unavailable to plant, P-solubilizing bacteria are important for plant nutrition. N_2_-fixing and P-solubilizing bacteria play an important role by increasing N and P uptake by the plants, and playing a significant role as plant growth promoting rhizobacteria (PGPR) in the biofertilization of crops. Plant growth-promoting rhizobacteria (PGPR) are able to exert a beneficial action upon plant growth by nitrogen fixation and P-solubilization [38]. There are reports of phosphate solubilising *Exiguobacterium sp. *having the potential to be used as a bioinoculants for paddy under alkaline soil conditions [39]. *Bacillus species *are well known for their siderophore production.* B. megaterium *produces schizokinen a derivatives of citrates (N-hydroxyl-L-acetyl ornithine citrate) [40]. *B. subtilis* produces a hexadentate siderophore bacillibactin and is structurally similar to the better known enterobactin of gram-negative bacteria such as *Escherichia coli *[41]. The ability to utilize the siderophores of another organism is of great selective advantage during iron-limited growth in the presence of a variety of competing microorganisms. Isolates must persist in the soil in the absence of the respective host plant, and this is a function of its competitiveness with other soil organisms for available nutrients. *Bradyrhizobium japonicum* USDA 110 and 61A152 can utilize the hydroxamate-type siderophores ferrichrome and rhodotorulate in addition to ferric citrate to overcome iron starvation. These strains can also utilize the pyoverdin type siderophore pseudobactin st3 [42]. Field tests performed with *B. japonicum *61A152 have consistently shown to give high yields of soybean [43], and this led to the speculation that its iron-scavenging property may be responsible for its competitive survival in rhizosphere. Assuming that these interactions occur in the rhizosphere, siderophore production, and utilization of siderophore produced by other organisms in the soil should play an important role in competition. Thus it was interesting to see whether the siderophores produced by the NAB and the rhizospheric isolates were being cross-utilized by the *S. meliloti*. Our earlier studies have shown rhizobial growth stimulation in presence of exogenously supplied siderophores that it is able to utilize [28] and we have shown that rhizobial strains when transformed with the ferrichrome siderophore receptor gene caused growth stimulation in the presence of Fe^3+^ [44] and also caused the plant growth promotion under both sterile and nonsterile soil conditions in lab [45]; we have also demonstrated the plant growth promoting effect of the siderophore produced by the *Bacilli sp*. on the rhizobial bioinoculants strains of* C. cajan* [26]. There are studies indicating a close correlation between leaf chlorophyll content and leaf N content [46]. Increased chlorophyll levels of the plant leaves inoculated with the NAB indicates efficient nitrogen fixation occurring in the nodules. 

In conclusion, the results of this study revealed that since M2N2c and B1N2b showed most of the plant growth promoting characteristics including IAA production, antifungal activity, phosphate solubilization, protease production, and so forth, and also *in-planta *coinoculation studies with standard rhizobial strain showed beneficial effect on overall plant growth. Thus these two strains may be used as a potent biofertilizer in combination with the rhizobial biofertilizer used for *Fenugreek.* Further characterization of this strain may help in providing the interesting results. 

## Figures and Tables

**Table 1:** Plant growth-promoting characteristics of the Fenugreek NAB and Rhizospheric isolates.

Sr no.	Isolates	Cell wall characteristics	Siderophore (C) (*μ*g/mL)*	Siderophore (H) (*μ*g/mL)^#^	IAA (*μ*g/mL)	Phosphate solubilization	Antifungal activity
1	M2EP1	−ve, CB^a^	+^ b ^ (449.5)^ c ^	+ (60)^ c ^	+ (43.68)^ c ^	—	+ A^a^
2	M1N1	−ve, CB	+ (414.7)	+ (84)	+ (19.36)	—	+ A
3	M2N2b	−ve, CB	+ (403.1)	+ (64)	+ (20.64)	—	+ A
4	M2N2a	+ve B	+ (350.9)	—	+ (23.68)	—	+ A
5	M2N2c	+ve B	+ (907.7)	+ (39.2)	+ (31.68)	+	+ A
6	M1EP1	−ve, CB	+ (321.9)	+ (36.4)	+ (28.32)	—	+ (A, F)
7	M2N1a	−ve, CB	+ (287.1)	+ (42)	+ (13.56)	—	+ A
8	M2N1b	−ve, CB	+ (327.8)	+ (136.2)	+ (25.44)	—	+ A
9	M2EP2	+ve B	+ (501.7)	+ (78.4)	+ (62.64)	—	+ A
10	M2EP3	−ve, CB	+ (298.7)	—	+ (21.28)	+	+ A
11	M1N3	+ve B	+ (284.2)	—	+ (30.4)	—	+ A
12	M2N2d	−ve, CB	+ (507.5)	—	+ (21.28)	—	+ A
13	M1N2	−ve, CB	+ (246.5)	+ (56)	+ (29.76)	—	+ A
14	B1N1	+ve C	+ (232)	+ (53.2)	+ (20.8)	—	+ A
15	B1N2a	−ve, CB	+ (362.5)	—	+ (28.8)	—	+ A
16	B1N2b	+ve B	+ (205.9)	+ (50.4)	+ (21.28)	+	+ A
17	B1N3	−ve, CB	+ (481.4)	+ (103.6)	+ (29.28)	—	+A

				Rhizospheric isolate			

18	C1	+ve B	+ (301.6)	+ (47.6)	+ (20.64)	—	—
19	C2	+ve B	—	+ (156.8)	+ (36.96)	++	—
20	C3	+ve B	+ (284.2)	+ (44.8)	+ (20.96)	+++	—
21	R1	+ve B	+ (394.9)	+ (106.4)	+ (34.72)	++	—
22	R2	−ve, CB	+ (214.6)	+ (100.8)	+ (33.44)	—	—
23	R3	+ve B	+ (524.6)	+ (132.2)	+ (33.84)	+++	+ R
24	R4	+ve B	+ (261)	+ (154)	+ (41.12)	++	—

^a^CB:* coccobacilli, *B:* bacilli and *C:* cocci*, A: *Alternaria*; F: *Fusarium* and R: *Rhizoctonia*; ^b^+ = production; ^c^figures indicated in bracket shows the concentration; *Catecholate siderophore was estimated by Arnow's method [28], ^#^hydroxamate siderophore was estimated by Gibson and Magrath method [28].

## References

[1] Antoun H, Kloepper JW, Brenner S, Miller JF (2001). Plant growth promoting rhizobacteria. *Encyclopedia of Genetics*.

[2] Zaidi A, Khan MS, Ahemad M, Oves M (2009). Plant growth promotion by phosphate solubilizing bacteria. *Acta Microbiologica et Immunologica Hungarica*.

[3] Glick BR (1995). The enhancement of plant growth by free-living bacteria. *The Canadian Journal of Microbiology*.

[4] Kloepper JW, Metting FB (1993). Plant growth-promoting rhizobacteria as biological control agents. *Soil Microbial Ecology-Applications in Agricultural and Environmental Management*.

[5] Parmar N, Dadarwal KR (1999). Stimulation of nitrogen fixation and induction of flavonoid-like compounds by rhizobacteria. *Journal of Applied Microbiology*.

[6] Bolton H, Elliott LF, Turco RF, Kennedy AC (1990). Rhizoplane colonization of pea seedlings by *Rhizobium leguminosarum* and a deleterious root colonizing *Pseudomonas spp.* and effects on plant growth. *Plant and Soil*.

[7] Grimes HD, Mount MS (1984). Influence oF *Pseudomonas putida* on nodulation of *Phaseolus vulgaris*. *Soil Biology and Biochemistry*.

[8] Polonenko DR, Scher FM, Kloepper JW, Singleton CA, Laliberte M, Zaleska I (1987). Effects of root colonizing bacteria on nodulation of soyabean roots by *Bradyrhizobium japonicum*. *The Canadian Journal of Microbiology*.

[9] Yaholom E, Okon Y, Dovrat A (1988). Early nodulation in legumes inoculated with Azospirillum and Rhizobium. *Symbiosis*.

[10] Sturz AV, Christie BR, Nowak J (2000). Bacterial endophytes: potential role in developing sustainable systems of crop production. *Critical Reviews in Plant Sciences*.

[11] Kobayashi DY, Palumbo JD, Bacon CW, White JF (2000). Bacterial endophytes and their effects on plants and uses agriculture. *Microbial endophytes*.

[12] Hallmann JA, Quadt-Hallmann A, Mahaffee WF, Kloepper JW (1997). Bacterial endophytes in agricultural crops. *The Canadian Journal of Microbiology*.

[13] Sturz AV, Christie BR, Matheson BG, Nowak J (1997). Biodiversity of endophytic bacteria which colonize red clover nodules, roots, stems and foliage and their influence on host growth. *Biology and Fertility of Soils*.

[14] Manninger E, Antal M (1970). Rhizobium and other bacteria in the root nodules of leguminous plants. I. Sterilization of the surface of root nodules of Soja max. *Zentralbl Bakteriol Parasitenkd Infektionskr Hygeine*.

[15] Gagne S, Richard C, Roussean H, Antoun H (1987). Xylem-residing bacteria in alfalfa roots. *The Canadian Journal of Microbiology*.

[16] Oehrle NW, Karr DB, Kremer RJ, Emerich DW (2000). Enhanced attachment of *Bradyrhizobium japonicum* to soybean through reduced root colonization of internally seedborne microorganisms. *The Canadian Journal of Microbiology*.

[17] Bai Y, D’Aoust F, Smith DL, Driscoll BT (2002). Isolation of plant-growth-promoting *Bacillus* strains from soybean root nodules. *The Canadian Journal of Microbiology*.

[18] Bai Y, Zhou X, Smith DL (2003). Crop ecology, management and quality: enhanced soybean plant growth resulting from coinoculation of *Bacillus* strains with *Bradyrhizobium japonicum*. *Crop Science*.

[19] Burns TA, Bishop PE, Israel DW (1981). Enhanced nodulation of leguminous plant roots by mixed cultures of *Azotobacter* vinelandi and damping-off of tomato by *Pseudomonas aeruginosa* 7NSK2. *Applied and Environmental Microbiology*.

[20] Srinivasan M, Petersen DJ, Holl FB (1996). Influence of indoleacetic acid-producing Bacillus isolates on the nodulation of *Phaseolus vulgaris* by Rhizobium etli under gnotobiotic conditions. *The Canadian Journal of Microbiology*.

[21] Chanway CP, Hynes RK, Nelson LM (1989). Plant growth-promoting rhizobacteria: effects on growth and nitrogen fixation of lentil (*Lens esculenta* Moench) and pea (*Pisum sativum* L.). *Soil Biology and Biochemistry*.

[22] Zhang F, Dashti N, Hynes RK, Smith DL (1996). Plant growth promoting rhizobacteria and soybean [*Glycine max* (L.) Merr.] nodulation and nitrogen fixation at suboptimal root zone temperatures. *Annals of Botany*.

[23] Liu ZL, Sinclair JB (1993). Colonization of soybean roots by *Bacillus megaterium* B 153-2-2. *Soil Biology and Biochemistry*.

[24] Sturz AV (1995). The role of endophytic bacteria during seed piece decay and potato tuberization. *Plant and Soil*.

[25] Vincent JM (1970). *A Manual for the Practical Study of Root Nodule Bacteria*.

[26] Rajendran G, Singh F, Desai AJ, Archana G (2008). Enhanced growth and nodulation of pigeon pea by co-inoculation of Bacillus strains along with *Rhizobium* sp.. *Bioreource Technology*.

[27] Gordon SA, Weber RP (1951). Colorimetric estimation of indoleacetic acid. *Plant Physiology*.

[28] Khan A, Geetha R, Akolkar A, Pandya A, Archana G, Desai AJ (2006). Differential cross-utilization of heterologous siderophores by nodule bacteria of *Cajanus cajan* and its possible role in growth under iron-limited conditions. *Applied Soil Ecology*.

[29] Graan T, Ort DR (1984). Quantitation of the rapid electron donors to P700, the functional plastoquinone pool, and the ratio of the photosystems in spinach chloroplasts. *Journal of Biological Chemistry*.

[30] Ryle GJ, Arnott RA, Powell CE (1981). Distribution of dry weight between root and shoot in white clover dependent on N_2_ fixation or utilizing abundant nitrate nitrogen. *Plant and Soil*.

[31] Barea JM, Pozo MJ, Azcón R, Azcón-Aguilar C (2005). Microbial co-operation in the rhizosphere. *Journal of Experimental Botany*.

[32] Bai Y, Souleimanov A, Smith DL (2002). An inducible activator produced by a *Serratia proteamaculans* strain and its soybean growth-promoting activity under greenhouse conditions. *Journal of Experimental Botany*.

[33] Araujo WL, Maccheroni W, Aguilar-Vildoso CI, Barroso PAV, Saridakis HO, Azevedo JL (2001). Variability and interactions between endophytic bacteria and fungi isolated from leaf tissues of citrus rootstocks. *The Canadian Journal of Microbiology*.

[34] Misaghi IJ, Donndelinger CR (1990). Endophytic bacteria in symptom-free cotton plants. *Phytopathology*.

[35] Rasche F, Trondl R, Naglreiter C, Reichenauer TG, Sessitsch A (2006). Chilling and cultivar type affect the diversity of bacterial endophytes colonizing sweet pepper (*Capsicum anuum* L.). *The Canadian Journal of Microbiology*.

[36] de Aguilar CA, Barea JM (1978). Effect of interactions between different culture fractions of phosphobacteria and *Rhizobium* on mycorrhizal infection, growth and nodulation of Medicago sativa. *The Canadian Journal of Microbiology*.

[37] Manero FJ, Probanza A, Ramos B, Colón Flores JJ, Lucas García JA (2003). Effects of culture filtrates of rhizobacteria isolated from wild lupine on germination, growth, and biological nitrogen fixation of lupine seedlings. *Journal of Plant Nutrition*.

[38] Zaidi A, Mohammad S (2006). Co-inoculation effects of phosphate solubilizing micro- organisms and glomus fasciculatum on green gram bradyrhizobium symbiosis. *Agricultural Science*.

[39] Kumar A, Bhargava P, Rai LC (2010). Isolation and molecular characterization of phosphate solubilizing *Enterobacter* and *Exiguobacterium* species from paddy fields of eastern Uttar Pradesh, India. *African Journal of Microbiology Research*.

[40] Byers BR, Powell MV, Lankford CE (1967). Iron-chelating hydroxamic acid (schizokinen) active in initiation of cell division in *Bacillus megaterium*. *Journal of Bacteriology*.

[41] Ollinger J, Song KB, Antelmann H, Hecker M, Helmann JD (2006). Role of the Fur regulon in iron transport in Bacillus subtilis. *Journal of Bacteriology*.

[42] Plessner O, Klapatch T, Guerinot ML (1993). Siderophore utilization by *Bradyrhizobium japonicum*. *Applied and Environmental Microbiology*.

[43] Hume DJ, Shelp BJ (1990). Superior performance of the hup- *Bradyrhizobium japonicum* strains 532C in Ontario soybean field trials. *The Canadian Journal of Plant Science*.

[44] Rajendran G, Mistry S, Desai AJ, Archana G (2007). Functional expression of *E.coli fhuA* gene in Rhizobium spp. of *Cajanus cajan* provides growth advantage in presence of Fe3+: ferrichrome as iron source. *Archives of Microbiology*.

[45] Geetha R, Desai AJ, Archana G (2009). Effect of the expression of *Escherichia coli fhuA* gene in *Rhizobium* sp. IC3123 and ST1 in planta: its role in increased nodule occupancy and function in pigeon pea. *Applied Soil Ecology*.

[46] Henriksson E, Pearson LC (1981). Nitrogen fixation rate and chlorophyll content of the lichen *Peltigera canina* exposed to sulfur dioxide. *The American Journal of Botany*.

